# First-in-human quantitative [¹⁵O]H₂O PET imaging of tumour blood flow at rest and during exercise in lymphoma patients

**DOI:** 10.2340/1651-226X.2026.45780

**Published:** 2026-06-24

**Authors:** Milla Perros, Tiia Koivula, Salla Lempiäinen, Tuula Tolvanen, Anna Kirjavainen, Laura Nummijärvi, Carl-Johan Sundberg, Helene Rundqvist, Heikki Minn, Kari Kalliokoski, Ilkka H.A. Heinonen

**Affiliations:** a Turku PET Centre, University of Turku and Turku University Hospital, and Åbo Akademi, Turku, Finland; b Department of Oncology and Radiotherapy, Turku University Hospital, Turku, Finland; c Department of Physiology and Pharmacology, Karolinska Institutet, Stockholm, Sweden; d Department of Laboratory Medicine, Karolinska Institutet, Stockholm, Sweden; e Department of Biomedical Engineering, Huazhong University of Science and Technology, Wuhan, China; f Department of Medical Physics, Turku University Hospital, Turku, Finland; g Department of Radiology, Turku University Hospital, Turku, Finland; h The UKK Institute for Health Promotion Research, Tampere, Finland

**Keywords:** Positron-emission tomography, lymphoma, tumour blood supply, exercise, oncology

## Abstract

**Background and purpose:**

Although exercise is recommended for cancer patients, its acute effects on tumour blood flow (TBF) have not been quantified in humans. As TBF may influence tumour progression and treatment efficacy, we assessed its circulatory responses in malignant lymphoma during exercise using dynamic PET imaging.

**Patient/material and methods:**

Eight patients with Hodgkin or non-Hodgkin lymphoma underwent thoracic [¹⁵O]H₂O positron emission tomography/computed tomography (PET/CT) at rest and during 10 min of supine cycling (Borg RPE 11–16). TBF and its heterogeneity, tumour blood volume (TBV), mean transit time (MTT), and vascular resistance were quantified in eight index and three secondary tumours.

**Results:**

Baseline TBF in index tumours was high (mean 57.6 mL/dL/min; range 30.3–105.0). During exercise, TBF (mean 48.1 ± 16.1 mL/dL/min), its heterogeneity and MTT did not change significantly. However, TBV decreased (*p* = 0.038), and vascular resistance tended to increase (*p* = 0.055). TBF change correlated positively with age (*r* = 0.73, *p* = 0.04) and negatively with tumour volume (*r* = –0.67, *p* = 0.02), but not with heart rate or power output. Secondary tumours showed similar exercise responses, with lower absolute TBF (*p* = 0.02).

**Interpretation:**

This study demonstrates the feasibility of using [¹⁵O]H₂O PET/CT to quantify TBF in lymphoma patients in real time during acute exercise. Baseline TBF is remarkably high in lymphoma tumours, and responses to acute exercise are variable, tending to decline in younger patients and larger tumours, while vascular resistance tended to increase.

## Introduction

Cancer is one of the leading causes of death worldwide and a major global public health challenge. Despite advances in screening, prevention, early diagnosis, and treatment, the global burden of cancer continues to rise, with an estimated 20 million new cancer cases and 9.7 million cancer deaths in 2022 [1]. Lymphomas are a heterogeneous group of lymphoid malignancies broadly classified into Hodgkin lymphoma (HL) and non-Hodgkin lymphoma (NHL) [2].

Lifestyle factors, including physical inactivity, play a significant role in cancer development and progression. Physical inactivity has reached alarming levels globally, with nearly one third of adults worldwide failing to meet the World Health Organization’s (WHO) recommendations for physical activity [3]. There is strong evidence that regular physical activity reduces the risk of several major cancer types, including colorectal and breast cancer, and emerging evidence suggests that it is also associated with improved survival outcomes [4–7]. In addition to its role in primary prevention, physical activity is increasingly recognised as a supportive strategy in cancer care, with growing evidence linking it to improved prognosis across multiple cancer types [8–10]. Exercise is a feasible and effective means of complementary treatment in lymphoma patients [11].

Preclinical studies suggest that physical activity may slow tumour progression and improve outcomes by modulating tumour growth, metastatic potential, metabolism, microenvironment, and immunology [12, 13]. Aerobic exercise training in animal models of prostate cancer has been shown to increase the mean number of perfused tumour vessels and perfused tumour area [14] and reduce tumour hypoxia [15] compared to control animals (from 3.0% of perfused area of total tumour to 7.4% of perfused area) [14]. This effect has been attributed to normalisation of the tortuous, leaky tumour vasculature, which despite strong angiogenic drive results in poor perfusion [14]; however, exercise training did not alter vessel contractile function [15]. In similar prostate cancer models, acute exercise did not alter blood flow to ectopic (subcutaneous) tumours, but increased the blood flow of orthotopic tumours by approximately 200% when measured with infusion of radiolabelled microspheres, indicating that tumour blood flow (TBF) is dependent on the location of the tumour and host tissue haemodynamics [16], and partly dependent on an attenuation of sympathetic vasoconstriction in preclinical models [14–16]. Moreover, exercise-induced increases in cardiac output and blood pressure likely elevate perfusion pressure within experimental tumours [17], contributing to changes in perfusion dynamics in tumour models. These haemodynamic changes can alter TBF and oxygenation, critical determinants of treatment efficacy, as blood flow of solid tumours is often poor, and together with hypoxia, is known to impair the response to most oncological therapies [18]. Heterogeneous TBF is a major contributor to hypoxia, and preclinical evidence suggests that exercise may reduce TBF heterogeneity and thereby improve tumour oxygenation [13, 14, 19]. In contrast, the direct mechanistic and clinical effects of acute exercise on human tumours remain largely unknown.

To assess whether these preclinical findings translate to humans – particularly to lymphoma patients – we conducted this study. Specifically, we investigated acute exercise-induced changes in TBF and its heterogeneity using [¹⁵O]H₂O PET/CT imaging in patients with HL or NHL before the onset of oncological treatment. We hypothesised that, consistent with preclinical data, TBF would increase during acute exercise.

## Patients/material and methods

This prospective, single-arm clinical study was conducted at the Turku PET Centre, Turku, Finland (ClinicalTrials.gov ID: NCT03987724). The study protocol was approved by the Ethics Committee of the Hospital District of Southwestern Finland. All participants provided written informed consent prior to enrolment. The study adhered to the principles of the Declaration of Helsinki and Good Clinical Practice guidelines.

### Patient population

Eight patients (six men, two women) with histologically confirmed HL or NHL, referred for oncological treatment at Turku University Hospital, were enrolled. Eligible patients were ≥18 years of age, had newly diagnosed (*n* = 5) or recurrent (*n* = 3) disease, and presented with tumour involvement in the neck and/or thorax. Staging was performed using whole-body CT (*n* = 3) or [¹⁸F]FDG PET/CT (*n* = 5), according to institutional protocols.

Exclusion criteria were abnormal fatigue, anaemia, or disease-related physical impairment; inability to comprehend written Finnish or any chronic condition posing risk or interfering with study procedures. Patients were selected based on physical fitness and favourable tumour locations for thoracic PET imaging. All HL cases (*n* = 3) were of the nodular sclerosis subtype. Among the NHL cases, two patients (Nos. 1 and 2) had diffuse large B-cell lymphoma, two (Nos. 3 and 5) had low-grade follicular lymphoma, and one (No. 8) had small lymphocytic lymphoma. PET imaging was performed prior to the initiation of oncological therapy. The average age of the patients was 54 years (range: 20–69 years), and their mean body mass index (BMI) was 26.6 kg/m² (range: 21.1–34.3 kg/m²). Patients’ characteristics are summarised in Table 1.

**Table 1:** Clinical and demographic characteristics of the study participants.

No.	Sex	Age	BMI (kg/m²)	Histologic type	Ann Arbor stage	IPI/IPS score*	Charlson Comorbidity index**	Index tumour volume (cm^3^)
1	M	60–64	34	Diffuse large B-cell lymphoma	IA	2	4	6.2
2	M	60–64	27	Diffuse large B-cell lymphoma	IVA	4	6	2.2
3	F	65–69	24	Follicular lymphoma	IIA	1	4	9.2
4	M	20–24	25	Hodgkin’s lymphoma	IVB	3	0	1332.7
5	M	65–69	27	Follicular lymphoma	IIIA	3	3	3.1
6	M	50–54	25	Hodgkin’s lymphoma	IIB	4	3	29.3
7	M	20–24	21	Hodgkin’s lymphoma	IIA	3	0	409.6
8	F	65–69	28	Small lymphocytic lymphoma	IIIA	0	2	2.5

BMI: body mass index; IPI: International Prognostic Index; IPS: International Prognostic Score; CCI: Charlson Comorbidity Index.

Patients with histologically confirmed lymphoma (*n* = 8) are described by sex (M, male, F, female), age group, BMI, histologic subtype, Ann Arbor stage, IPI or IPS, CCI and index tumour volume (cm^3^). Age is presented in 5-year ranges to ensure patient confidentiality.

### Exercise imaging protocol and physiological monitoring

**Figure 1:**
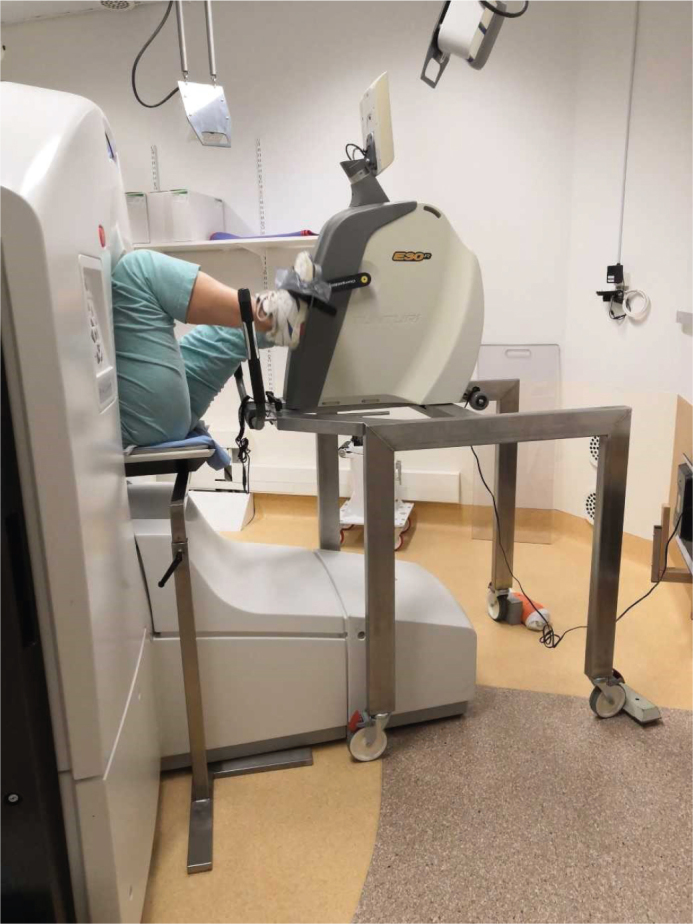
Supine bicycle exercise setup during [¹⁵O]H₂O PET imaging. To assess the effects of acute exercise on TBF and its heterogeneity, patients performed a 10-min session of supine cycling on a modified ergometer (Tunturi E30R; Tunturi New Fitness B.V., Almere, The Netherlands) during dynamic PET acquisition. Exercise intensity was tailored individually based on pre-study evaluation to ensure a sustainable pedalling rhythm. Heart rate (HR) and perceived exertion were monitored using a pulse oximeter (Palmsat 2500; Nonin, USA) and the Borg scale, respectively. Workload was recorded in watts.

To assess the effects of acute exercise on TBF and its heterogeneity, patients performed a 10-min session of supine cycling on a modified ergometer (Tunturi E30R, Tunturi New Fitness B.V., Almere, The Netherlands) during dynamic PET acquisition (Figure 1). The exercise protocol, previously utilised at the Turku PET Centre [20–22], was incorporated into the clinical imaging schedule without delaying the start of treatment or introducing additional interventions. Participants were instructed to abstain from vigorous physical activity, alcohol, and caffeine for ≥ 24 h prior to imaging. Exercise intensity was not standardised to a fixed absolute workload or percentage of maximal capacity to be similar in all patients, but was individually adjusted based on perceived exertion (Borg RPE 11–16) to ensure feasibility and tolerability in this patient population. Heart rate (HR) and perceived exertion were monitored using a pulse oximeter (Palmsat 2500, Nonin, USA) and the Borg scale, respectively. Workload was recorded in watts.

Systemic haemodynamic parameters – HR, systolic and diastolic blood pressure (Apteq AE701f, Rossmax Swiss GmbH, Switzerland), and arterial oxygen saturation – were monitored continuously. The rate–pressure product (RPP) was calculated as HR × systolic blood pressure. Mean arterial pressure (MAP) was calculated as [(2 × diastolic) + systolic] / 3. The predicted maximal HR (bpm) was calculated as 220 − age. TBF-related variables were assessed at rest and during exercise, including tumour blood volume (TBV), mean transit time (MTT, mL/dL·s), and tumour vascular resistance (mmHg·dL^–^¹·mL^–^¹·min^–^¹), along with systemic parameters such as HR, MAP, RPP, and oxygen saturation.

### Radiotracer production

[¹⁵O]H₂O was synthesised at the Turku PET Centre using established protocol [23]. Good Manufacturing Practice (GMP) procedures were followed to verify the radiochemical purity, pH, and radioactivity of the [¹⁵O]H₂O bolus.

### PET/CT imaging protocol

Imaging was performed using either a GE Discovery 690 or GE Discovery MI PET/CT scanner (General Electric Medical Systems, GEMS, Milwaukee, WI, USA), depending on scanner availability [24]. A venous catheter was inserted into a forearm vein for tracer administration. Patients were positioned supine with feet secured to the ergometer and arms elevated above the head for stability (Figure 1). A low-dose CT scan was acquired for attenuation correction, and standard corrections for attenuation, scatter, and decay were applied. Dynamic PET images acquired with the Discovery 690 scanner were reconstructed using the GE VUEPoint FX SharpIR (VPFXS) algorithm, whereas images acquired with the Discovery MI scanner were reconstructed using the GE VUEPoint HD SharpIR (VPHDS) algorithm, with 6 iterations and 17 subsets, using a 256 × 256 matrix and a 70 cm display field of view. A bolus of ~700 MBq [¹⁵O]H₂O was administered intravenously, followed by a 6-min dynamic PET acquisition of the thorax and lower neck at rest. After a decay period, the patient commenced supine cycling, and a second 6-min dynamic scan was initiated during the exercise session (start time: 4 min after exercise onset, thus steady-state exercise).

### Region-of-interest definition

**Figure 2:**
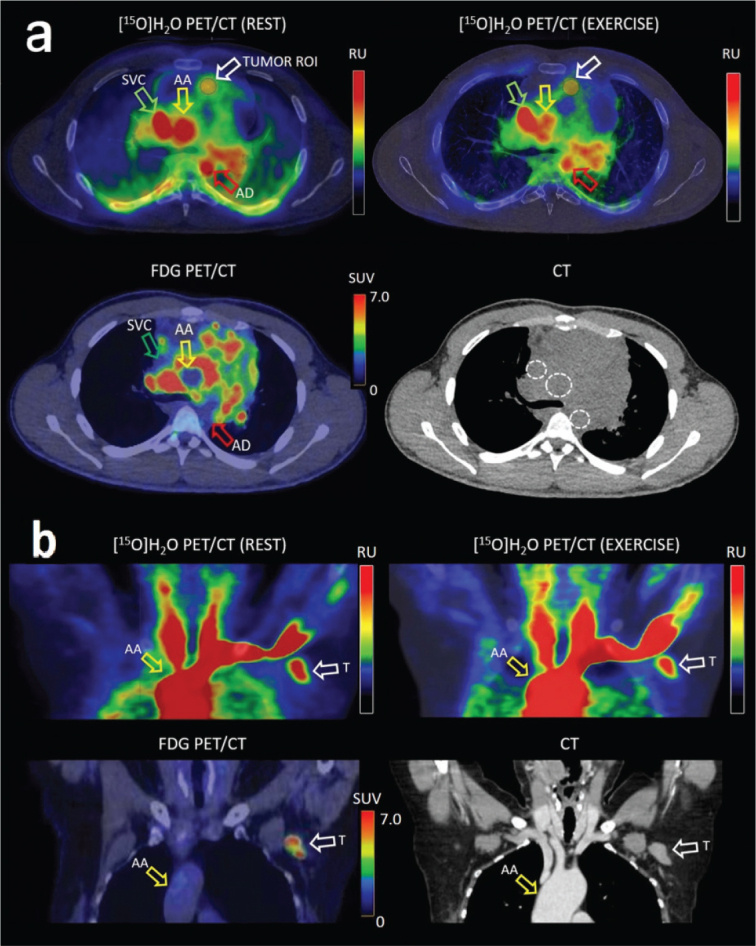
Two patient cases illustrating tumour identification and analyses. (A) Axial [¹⁵O]H₂O PET/CT summed radioactivity images (0–90 s) at rest and during acute exercise in a 20-year-old man with Hodgkin’s lymphoma. Images were reconstructed using the scanner-specific GE reconstruction algorithm, as described in the Methods. This patient (No. 4) presented with dyspnoea due to a bulky mediastinal tumour (index tumour) showing highly heterogeneous perfusion and metabolism. The retrosternal part of the tumour with the highest regional TBF and the corresponding ROI (green circle) are indicated with an open white arrow. The superior vena cava (SVC), ascending aorta (AA), and descending aorta (AD) are marked with green, yellow, and red open arrows, respectively. Colour scale bars indicate relative uptake (RU). The diagnostic [¹⁸F]FDG PET/CT and low-dose CT scans highlight the large size of the tumour. On CT, the great vessels are outlined with dashed white circles. SUV, standardised uptake value; ROI, region of interest; T, tumour. (B) Coronal [¹⁵O]H₂O PET/CT summed radioactivity images (0–90 s) at rest and during acute exercise in a 69-year-old woman with follicular lymphoma. Images were reconstructed using the scanner-specific GE reconstruction algorithm, as described in the Methods. She had previously achieved complete remission following chemotherapy and rituximab for diffuse large B-cell lymphoma and now presented with recurrence in the left axilla confirmed by diagnostic [¹⁸F]FDG PET/CT and contrast-enhanced CT. The tumour (T) is clearly visible on all coronal scans (white arrow), together with the ascending aorta (AA). Colour scale bars indicate relative uptake (RU) and standardised uptake value (SUV).

An experienced specialist in diagnostic radiology first reviewed the diagnostic CT or [^18^F]FDG PET/CT scans in each patient and selected a main lymphoma nodule as the index tumour for TBF measurement. Three patients had an additional secondary tumour conveniently located in the same field-of-view available for analysis. All 11 tumours were identified visually from the experimental [^15^O]H_2_O scans using low-dose CT guidance, tracer uptake patterns, and prior diagnostic imaging. Differences in patient positioning precluded direct co-registration of diagnostic and experimental CT or PET/CT scans. Regions of interest (ROIs) were then manually delineated and drawn using the Carimas software platform [25] (version 2.10, https://turkupetcentre.fi/carimas/academic/features/) in all cross-sectional planes with visible tracer accumulation and automatically summed to create a 3D ROI representing the TBF-activated tumour volume. For exercise images, the resting ROIs were transferred and aligned to ensure anatomical consistency. No respiratory gating was performed, and therefore motion correction was not applied. In cases with large or irregular HL masses, the most representative region was selected for analysis (Figure 2).

### Input function and reference tissue

The ascending aorta was used as the source of the input function in seven patients; in one case (No. 6), the right common carotid artery was used due to tumour location. A circular ROI was manually drawn over the vessel lumen and applied consistently across rest and exercise scans. The same ROI location for input function was used for both rest and exercise measurements within each individual. The right deltoid muscle served as a reference tissue representing resting muscle blood flow.

### Blood flow calculation, its heterogeneity and tumour vascular resistance

TBF based on time-activity curves was quantified using a previously validated [¹⁵O]H₂O kinetic model (http://www.turkupetcentre.net/petanalysis/model_radiowater.html).

Due to the limited spatial resolution of PET-[¹⁵O]H₂O for assessment of spatial blood flow heterogeneity, summed radioactivity images (0–90 s) were used to assess spatial heterogeneity, assuming a linear correlation between tracer activity and blood flow. TBF heterogeneity was then defined as the voxel-wise coefficient of radioactivity variation (standard deviation [SD] within the ROI divided by mean radioactivity). Tumour vascular resistance was calculated as MAP divided by corresponding TBF.

### Statistical methods

Results are presented as mean ± SD and, where appropriate, median and range. Statistical analyses were performed using SAS, version 9.4 (SAS Institute Inc., Cary, NC, USA) and GraphPad Prism (version 8.0). Dependent Student’s t-test was used to compare rest and exercise values. For secondary analyses, mixed- effects models for repeated measures were applied to examine tumour, time, and tumour × time interaction effects. Correlations were evaluated using two-tailed Pearson’s correlation coefficient. A *p*-value <0.05 was considered statistically significant.

## Results

In total, 21 participants were contacted and found eligible in the screening phase. Of them, two women and six men (38%) volunteered for this study. Pedalling power of the exercise ranged from 20 to 90 watts (average 57 ± 25) between subjects. According to the subject´s own experience and their sense of fatigue, RPE ranged from 11 to 16 (average 13 ± 1) on the Borg scale (20 corresponds to maximal exhaustive effort). HR increased from rest (77 ± 14 bpm) to exercise (112 ± 20 bpm). The latter was 68 ± 14% of the age-predicted maximal HR. Also MAP (from 89 ± 9 mmHg to 104 ± 25 mmHg) and RPP (from 9,792 ± 1,934 bpm • mmHg to 16,642 ± 4,122 bpm • mmHg) increased by exercise. All of these physiological exertion measures increased from rest to exercise (*p* < 0.05). The index tumour volume showed a highly skewed distribution (median 7.7 cm³, range 2.2–1332.7; mean 224.3 ± 469.4 cm³, *n* = 8), reflecting substantial interindividual variability. The mean secondary tumour volume was 4.9 ± 3.1 cm³ (median 3.2 cm³, range 3.0–8.6; *n* = 3). Individual index tumour volumes are shown in Table 1.

**Figure 3:**
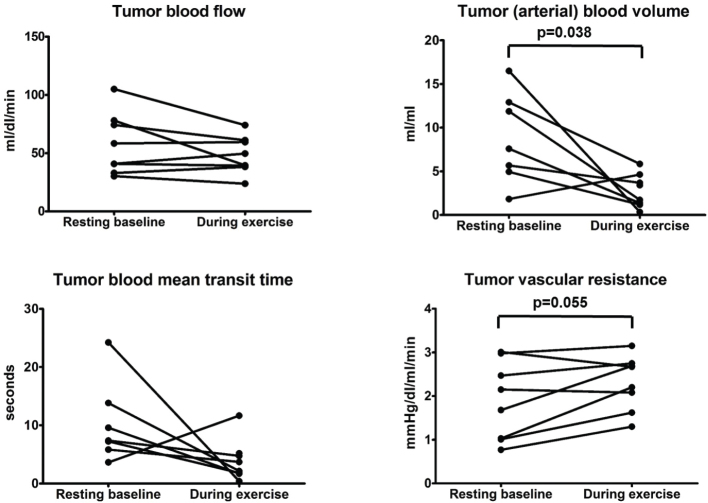
Tumour blood flow (TBF), tumour (arterial) blood volume (BV), tumour blood mean transit time (MTT), and tumour vascular resistance at resting baseline and during exercise. The resting baseline TBF in lymphoma index tumours was high (median 49.6 mL/dL/min, range 30.3–105.0; mean 57.6 ± 26.4 mL/dL/min), and during exercise TBF was 44.6 mL/dL/min (range 23.8–74.0; mean 48.1 ± 16.1 mL/dL/min).

**Figure 4:**
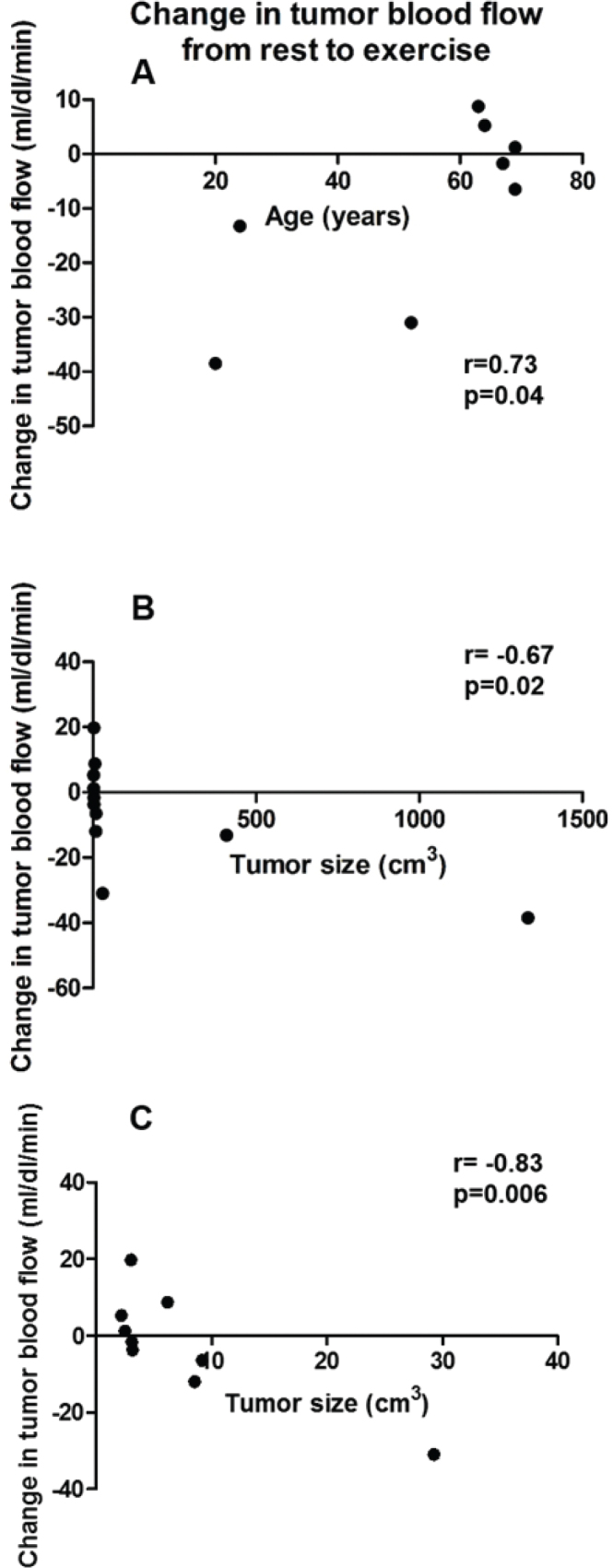
Correlations between the change in tumour blood flow (TBF) from rest to exercise and patient or tumour characteristics. (A) Exercise- induced change in TBF correlated positively with age (r = 0.73, p = 0.04), and (B) negatively with tumour volume (r = –0.67, p = 0.02). (C) When the two largest tumours were excluded, the negative correlation between tumour volume (cm³) and change in TBF remained significant and stronger (r = –0.83, p = 0.006).

Representative TBF PET radiowater images at rest and during exercise, along with corresponding diagnostic CT and [¹⁸F]FDG PET/CT scans of two patients, are shown in Figures 2A and 2B. The resting baseline TBF in lymphoma index tumours was high (median 49.6 mL/dL/min, range 30.3–105.0; mean 57.6 ± 26.4 mL/dL/min), and remained similar during exercise (median 44.6 mL/dL/min, range 23.8–74.0; mean 48.1 ± 16.1 mL/dL/min) (Figure 3). Mean TBF heterogeneity at rest was 36 ± 16% and during exercise 34 ± 13%. TBV was significantly (p = 0.038)reduced and tumour vascular resistance tended (*p* = 0.055) to increase (Figure 3). Exercise-induced change in TBF (Figure 4) correlated positively with age and negatively with the tumour volume, but not with any other exercise exertion measures such as power output or HR (data not shown). Tumour volume did not correlate with TBF at rest (*r* = 0.46, *p* = 0.15) or during exercise (*r* = 0.07, *p* = 0.84).

**Figure 5:**
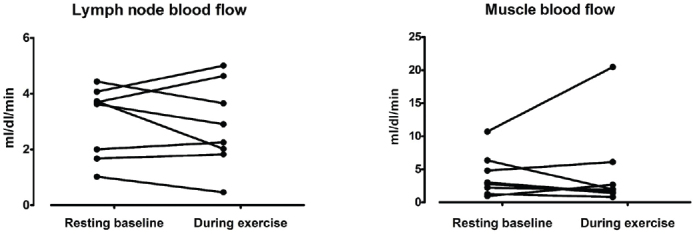
Blood flow in lymph nodes and skeletal muscle (m. deltoideus) at resting baseline and during exercise. There was no significant change in lymph node or (inactive) skeletal muscle blood flow from rest to exercise.

Inclusion of secondary tumours in the analyses revealed that the interaction of tumour × time was not significant in terms of TBF (*p* = 0.43), thus TBF responses did not depend on the index/location/size of the tumours. As expected, TBF was significantly lower (*p* = 0.02) in the three secondary tumours compared to eight index tumours, both at rest and during exercise (secondary TBF at rest 24.2 ± 6.9 mL/dL/min and in exercise 25.5 ± 11.3 mL/dL/min). Furthermore, vascular resistance tended (*p* = 0.08) to be higher in the secondary tumours than that in the index tumours, but no main or interaction effects were seen between the index versus secondary tumour BV and MTT variables (all *p*-values > 0.14, data not shown). Lymph node or inactive muscle (deltoid muscle in the image area) blood flow did not change from rest to exercise (Figure 5). Lymph node or muscle BV, blood MTT or vascular resistance did not change either from rest to exercise (data not shown).

## Discussion and conclusion

To the best of our knowledge, this is the first prospective clinical study to evaluate TBF during acute exercise in human cancer patients. Using [^15^O]H_2_O PET/CT, we demonstrated the feasibility of non-invasive, quantitative imaging of TBF in patients with lymphoma under physiological exercise stress. Although preclinical studies have suggested that aerobic exercise may enhance tumour perfusion [14–16, 26], our findings do not support these preclinical findings regarding TBF during acute exercise in human lymphoma. Instead, TBF responses in these patients were heterogeneous, with several patients demonstrating reduced TBF during exercise. These results underline the complexity of human tumour vascular regulation and the challenges in translating exercise-oncology findings from animal models to the clinical setting.

At rest, TBF in lymphoma tumours was relatively high, ranging from 30.3 to 105.0 mL/dL/min, with a mean value several-fold higher than that typically reported, for instance, in resting skeletal muscle (~2–4 mL/dL/min) [27]. Compared to solid tumours studied with [^15^O]H_2_O PET, such as prostate (16–26 mL/dL/min) [28], breast (30 mL/dL/min before therapy) [29], or head and neck cancers (30–65 mL/dL/min) [30], TBF in our lymphoma cohort appears notably high. Historical clearance-based studies have also described relatively high blood flow in lymphoma, with average values around 35 mL/dL/min [31], but our quantitative and validated values are thus even higher.

The most important question for the present investigation was whether TBF increases from rest to exercise, as suggested by preclinical studies [14, 15]. This is an important question to answer as it has been suggested and studied [32], based on only preclinical evidence, that chemotherapy should be administered during exercise, with the rationale that increased TBF during exercise would direct more chemotherapy to the tumour, leading to improved treatment responses. However, our results do not support this assumption in the context of acute exercise in lymphoma. While a few patients demonstrated increased TBF during exercise, most patients showed decreased TBF, whereas the mean group response did not differ from resting baseline values. Furthermore, the significant decrease in TBV during exercise, alongside a tendency for increased vascular resistance, may rather reflect exercise-induced functional vasoconstriction in the tumour during exercise, suggesting that (sympathetic) regulatory mechanisms may be well-functioning in patients.

Notably, the change in TBF during exercise correlated positively with age and negatively with tumour volume (Figure 4). Thus, older age and smaller tumour volume were associated with larger increases in TBF, whereas younger patients and those with larger tumours tended to show little change or even reductions, indicating that vascular reactivity is not uniform across patients and tumours. These associations may partly be interrelated, as the two youngest patients in this cohort were HL patients with the largest tumours. Owing to the small sample size, these findings should therefore be interpreted as exploratory. Such variability is consistent with prior findings showing that tumour vasculature differs markedly between patients and even within tumours, contributing to spatially heterogeneous perfusion and drug delivery [33]. Abnormal vessel architecture, elevated interstitial pressure, and stromal resistance are known to limit perfusion and may explain the reductions in TBF observed in some participants despite increased flow in systemic circulation. In contrast, no relationship was found between TBF changes and conventional markers of exertion, such as cycling power or HR. These dynamic responses contrast with the well-documented baseline vascular heterogeneity in lymphoma [34] and are consistent with the broader variability reported in exercise-oncology studies [35]. In this cohort, tumour MTT and perfusion heterogeneity remained unchanged during exercise, suggesting that exercise-induced blood flow alterations were likely due to limited vasodilatory capacity rather than intratumoural blood redistribution. Secondary tumours exhibited lower TBF than index tumours at both rest and exercise, with no interaction between tumour site and condition. No significant vascular changes were detected in reference tissues such as lymph nodes or skeletal muscle, indicating stable background perfusion. Other vascular parameters in secondary tumours, including BV and MTT, also showed no condition-dependent effects, although vascular resistance tended to be higher.

Some degree of methodological variability should also be considered when interpreting individual TBF responses. Although [¹⁵O]H₂O PET is a validated quantitative perfusion method, repeated dynamic measurements during exercise are susceptible to variability related to motion, ROI delineation, and image-derived input functions. Previous test–retest studies suggest repeatability coefficients in the range of approximately 30–40% [36–39]. Thus, modest individual changes should be interpreted cautiously. However, the concurrent decrease in TBV, tendency towards increased vascular resistance, and associations between TBF changes, age, and tumour volume suggest that the observed heterogeneity is unlikely to be explained by measurement variability alone.

The present findings differ from several preclinical studies in murine models, where acute exercise – particularly in orthotopic tumours – enhanced perfusion, reduced hypoxia, and improved drug delivery [14, 15]. In contrast, ectopic tumours such as subcutaneous implants often demonstrate blunted or contrasting responses, likely due to poor vascular integration and normal or even heightened susceptibility to sympathetic vasoconstriction [16]. Although human lymphomas are not ectopic in this sense, the limited perfusion response observed here may reflect similar physiological constraints, including aberrant vessel architecture, elevated interstitial pressure, and impaired vasoregulatory capacity [40, 41]. In cases where vasoregulatory mechanisms are preserved, acute exercise is likely to elicit functional vasoconstriction within the tumour, reducing TBF despite increased systemic circulation, which may partly explain our findings.

The absence of a systematic TBF increase raises questions regarding the timing and durability of exercise-induced vascular effects. Peak changes may occur after, rather than during, exercise, and repeated aerobic sessions may be required to induce vascular remodelling or normalisation, thereby improving oxygenation and treatment efficacy [18, 40]. The observed associations between TBF changes, age, and tumour volume suggest that both host and tumour characteristics influence vascular responsiveness, warranting further study into determinants such as autonomic nervous system regulation and microenvironmental heterogeneity [19, 42].

Despite the modest sample size (*n* = 8) and biological heterogeneity from the inclusion of both HL and NHL, this study demonstrates the technical feasibility of [¹⁵O]H₂O PET during exercise in a clinical setting. The generalisability of these findings to other cancer types remains uncertain. Lymphoma differs from many solid tumours in vascular architecture and microenvironment, and exercise-induced perfusion responses may therefore vary between tumour types and anatomical locations. These findings highlight the need for tumour-specific studies investigating haemodynamic responses to exercise.

In summary, this study is, to the best of our knowledge, the first to quantify TBF during acute exercise using validated [¹⁵O]H₂O PET/CT. The findings provide novel insights into lymphoma vascular behaviour under physiological stress and support further investigation of exercise as a modulator of tumour haemodynamics. However, the present results caution against assuming that tumour perfusion uniformly increases during acute exercise in human cancers.

### Strengths and limitations

This study has several important strengths. The study quantified TBF at rest and during acute exercise using validated dynamic [¹⁵O]H₂O PET/CT imaging. The protocol thus enabled real-time assessment of tumour perfusion under physiological exercise stress in a clinically feasible setting. In addition, the high sensitivity and spatial resolution of the method enabled detection of physiologically relevant perfusion patterns in both primary and secondary tumours.

The study also has limitations. The sample size was small and included both HL and NHL, limiting subgroup analyses and generalisability. Exercise intensity was individually adjusted rather than standardised to a fixed workload, which may have contributed to interindividual variability in haemodynamic responses. Respiratory motion, patient movement during exercise, and manual co-registration of ROIs between rest and exercise scans may also have affected perfusion estimates, particularly in smaller lesions. In addition, the current supine cycling protocol is most suitable for thoracic tumours and may not be directly applicable to other anatomical regions. Furthermore, [¹⁵O]H₂O PET quantifies total vascular volume rather than exclusively perfused vessels, which may influence absolute TBF estimates. In addition, repeated dynamic [¹⁵O]H₂O PET measurements during exercise are subject to methodological variability related to motion, ROI delineation, and image-derived input functions, and modest individual changes should therefore be interpreted cautiously in the light of previously reported repeatability coefficients.

## Conclusion

This study demonstrates the feasibility of using [¹⁵O]H₂O PET/CT to quantify TBF in lymphoma patients in real time not only at rest but also during acute exercise. The protocol provides a robust platform for future investigations into the effects of physical activity on tumour haemodynamics and its therapeutic relevance. Importantly, our findings caution against assuming that acute exercise consistently augments TBF in human cancers. The observed associations with age and tumour volume further highlight the role of host and tumour characteristics in modulating vascular responses. Future studies should explore whether repeated bouts of exercise or chronic training can induce vascular remodelling, improve blood flow and oxygenation, and enhance treatment efficacy in cancers other than lymphoma.

## Data Availability

The datasets generated and/or analysed during this study are available from the corresponding author on reasonable request.
